# 2345 An electronic roadmap to customized human research training plans

**DOI:** 10.1017/cts.2018.196

**Published:** 2018-11-21

**Authors:** Jennifer Maas, Megan Hoffman, Janet Shanedling, Jason Kadrmas, Trung Ngo, Jacob Johnson

**Affiliations:** CTSI, University of Minnesota

## Abstract

OBJECTIVES/SPECIFIC AIMS: To respond to the need for a simple tool to answer individual researchers questions: Exactly what training do I need to complete for my study and my role? Where can we go to find a comprehensive record of my research training? METHODS/STUDY POPULATION: Identify the factors that determine what training is required for each role (i.e., PI, coordinator, biostatistician) at the University, their role on the research study, type of funding, population being studied and responsibilities/duties on the research team. Develop an inventory of training required according to federal and local regulations and guidelines. Identify other related factors that ensure ongoing compliance for research professionals (i.e., medical licenses, CVs, immunizations, and credentials). Collaborate with programming professionals to explore and confirm the feasibility of such a Web site. Incorporate formal usability and pilot testing as part of the programming design process. Develop User Guide and Marketing and Launch plan for users and supervisors. Implement phased launch of the site with Google analytics, and evaluate the experience of phase I users. RESULTS/ANTICIPATED RESULTS: Three months user data and evaluation results demonstrated: 149 users created Training Roadmaps on the site. Users were from 67 different department codes, with the Department of Psychiatry the primary user. 20 users responded to a survey three months after launch. Research coordinators were the primary focus for phase I and represented almost half of the users. Survey respondents rated the site ease of use and clarity of the site as its greatest benefit. DISCUSSION/SIGNIFICANCE OF IMPACT: In September 2017, CTSI launched a new web-based training tool exclusively for University of Minnesota clinical research professionals who work with human participants, and their supervisors. The Human Research Training Web site is a free, easy-to-use tool to help identify and maintain the appropriate training, certification, credentials, and immunizations needed to perform University of Minnesota research with human participants. The Web site offers the University’s first systematic way to identify which research training is necessary for each research professional, and a system to track and maintain training compliance. Training records and information from the University of Minnesota’s central databases are securely integrated into this tool. Our Web site tool enhances research compliance. Any given study team member’s training requirements vary based on several criteria such as: role at the University, role on the research study, type of funding, population being studied and responsibilities/duties on the research study. The research training Web site generates required and optional training based on individuals’ responses to these questions. This Web site also links to the training, which decreases error in taking the wrong training. Furthermore, it provides completion data for research training and is a repository for vital study information such as: medical licenses, CVs, and credentials. Supervisors are able to view training and credentials. They are alerted when one of their employee’s licenses or certificates are about to expire. Uses-to-date and evaluation feedback have informed the need for a second phase of Web site enhancements. This site will reside in both the CTSI Web site and the HRPP Web site. A link will be sent to all new University research employees upon hiring. The Human Research Training Web site will likely have applicability to other universities in addition to the University of Minnesota.

